# Eco-Technological Evaluation of Natural Phytochemicals Potential Drug Molecules Against Main Protease: A Machine Learning Algorithm

**DOI:** 10.7759/cureus.57151

**Published:** 2024-03-28

**Authors:** Silambarasan Tamil Selvan, Mukesh Kumar Dharmalingam Jothinathan

**Affiliations:** 1 Center for Global Health Research, Saveetha Medical College and Hospitals, Saveetha Institute of Medical and Technical Sciences (SIMATS), Chennai, IND

**Keywords:** free energy binding, toxicity, density functional theory, molecular simulation, molecular docking, flavonoids, main protease, coronavirus

## Abstract

Introduction*: *The global viral pandemic has rapidly spread, leading to many individuals experiencing the infection. Coronaviruses (CoVs) are among many viral families that infect different types of mammals. They can spread to humans and cause gastrointestinal, neurological, and respiratory problems. The present investigation has discovered flavonoid compounds as promising molecular agents with potential antiviral activity against virus proteins, specifically main protease (Mpro).

Methodology: A comprehensive in silico screening of natural compounds derived from medicinal plants was performed in the present study. It included parameter assessments such as drug-likeness, pharmacokinetics, molecular docking, toxicity evaluations, bioavailability assessments, and molecular target exploration. In this systematic approach, the primary objective was to identify potential lead compounds. These phytochemicals were investigated as drug candidates to provide a detailed understanding of their molecular properties.

Results*: *The Mpro binding energy values were -10.637, -12.752, -7.813, -15.732, -6.449, -5.578, -8.037, and -8.52 kcal/mol for isoquercetin, narirutin, myricetin, hesperidin, silibinin, baicalein, taxifolin, and petunidin. Molecular simulations were conducted on two flavonoid compounds - hesperidin and narirutin - stable over 100 nanoseconds in the Coronavirus protein.

Conclusions: The computational study we conducted is promising, but to validate the action of these compounds, further experimental studies are needed, with a critical component of the research being the conduct of in vitro and in vivo experiments.

## Introduction

Coronaviruses (CoVs) are among many viral families that infect different types of mammals. They can spread to humans and cause gastrointestinal, neurological, and respiratory problems. Before this, two pandemic epidemics caused by the Middle East respiratory syndrome CoV (MERS-CoV) and SARS-CoV-2 seriously infected the human population [1]. Similar to SARS-CoV-2, which is currently responsible for deadly viral pneumonia and raising concerns about global health [2,3], the CoV has enormous viral RNA genomes and is characterized by positive-sense, single-stranded RNA viruses. The main protease (Mpro), a widely recognized CoV protein, has been identified as a potential therapeutic target. Mpro plays a crucial role in transcribing viral RNA, leading to the synthesis of polyproteins. Mpro, a widely recognized CoV protein, has been identified as a potential therapeutic target [4,5].

The South Indian medicinal plants contain various potential bioactive compounds, viz., flavonoids, polyphenols, therphiniods, phenolic acids, anthocyanins, and tannins [6-8]. The medicinal plant-derived bioactive compounds have demonstrated various pharmacological and biological activities, including anti-proliferative, anti-inflammatory, anti-atherogenic, anti-allergic, antimicrobial, immunomodulatory, and antiviral properties for human health [9,10]. They are also nontoxic, natural compounds that are friendly to the environment.

This study focused on identifying potential natural bioactive drug molecules from medicinal plant-derived flavonoid compounds. Compounds were used to investigate the suppression of corona viral protease and replication receptors, viz., Mpro protein using molecular docking, silicon toxicity, binding free energy calculations, molecular dynamics simulation, and density functional theory (DFT) analysis. The study opens further avenues for the development of functionally improved drug molecules.

## Materials and methods

Selection of ligand and preparation

The eight flavonoid ligand compounds were selected from various medicinal plants and the flavonoid compounds selected, viz., taxifolin, narirutin, hesperidin, baicalein, isoquercetin, myricetin, petunidin, and silibinin. The compound structures were derived from the PubChem database. The ligands were prepared and screened against the predicted CoV. The Mpro structure used Maestro v11 (Schrödinger, JSS University, Ooty) software. The design was developed with the Schrödinger protein preparation wizard. Protein optimization was performed at a neutral pH, and then the building structure with optimized liquid simulated force field potentials for all atoms was reduced. A 10 x 10 Å to 10 Å receptor grid was generated with Glide v7.1 (Schrödinger, New York, NY) in the Mpro-defined retention site residuals. The ligand was modified for chemical accuracy (protonation), stereochemical variations, ionization, neutral pH 7.0 ± 2.0, and energy using LigPrep modules [11].

Evaluation of silicon toxicity

The Swiss ADME online software was used to analyze the toxicity of the chosen flavonoid compounds, and the cytotoxicity, immunogenicity, hepatotoxicity, mutagenicity, and carcinogenicity toxicity parameters were evaluated. In this study, animal models were analyzed to identify the hazardous dose and reduce the number of models tested on animals [11].

Drug molecules and protein interaction analysis

The Maestro v11 software (Schrödinger, version 11) was used to do a molecular docking study of the active sites of the CoV Mpro protein with ligands. The polar H-atoms and Gasteiger charges were added to the protein amino acid atoms, and the appropriate protonation forms were assigned to the crystal Mpro protein structure. The protein structure's water (H2O) molecules were eliminated, and the protein's ligand and receptor bins (-0.28 to 0.55), outline bins (-0.22 to 0.44), and ligand and receptor sphere (1.3 to 1.55) were fixed. For Mpros, the outside of active sites remained unaltered, while partially active sites were kept flexible for the docking process. The compounds' competency docking score and binding hit were assessed and reported [11,12].

Molecular dynamics (MD) simulations

The Desmond v. 4.2 software program (Schrödinger, JSS University) examined the MD simulation evaluation to confirm prospective flavonoid compounds' ligand-receptor binding energy, interactions, and stability behavior. Explicit MD simulations were analyzed to run 100 ns MD simulations and neutralize water molecules. Ions were chosen to form solvating complexes. Energy minimization and periodic boundary conditions with an acceptable 1,000 kJ/mol/nm value were utilized. The outcomes of MD simulations of protein-ligand complexes were examined and documented [11].

Analysis of DFT 

Gaussian 09W software (Schrödinger, JSS University) was used to analyze the interactions between receivers and ligands in electronic structures were examined using DFT. The most occupied homo molecular orbital and the least occupied lowest unoccupied molecular orbital (LUMO), electron affinity, and electrophilicity indicator are the estimated parameters used to describe the electronic and structural characteristics of the five best-hit compounds. According to the study, molecular electrostatic possible surfaces (MEPs) were calculated using population analysis calculations and depicted using Gauss View 5.0. These parameters account for the magnitude of the ligand interactions at the binding site of CoV Mpro [11].

Analysis of binding free energy calculation

In the prime MMGBSA (molecular mechanics with generalized Born and surface area solvation) approach, the free energy binding of the ligand and protein was calculated. This method was used to show and guess the correlated free energy of binding (Δ*G*_bind_ ) for each group of ligand molecules to the protein. The docking poses were made as small as possible using the OPLS-AA2005 force field, and the Generalized Born/Surface Area (GB/SA) continuum solvent approach was utilized to determine the free energy of binding for the ligands. The MMGBSA model and Surface Generalized Born (SGB) design used Desmond software to perform the simulation analysis [13].

## Results

Selection and preparation of ligands

The eight flavonoid ligand compounds were selected from different medicinal plants. The molecular docking was analyzed for the chosen flavonoid ligands. The selected ligands were obtained in SDF format, and their two-dimensional (2D) structures were generated from the PubChem database. The ligand compound files in SDF format were converted to protein database (PDB) format using Discovery Studio software. The isoquercetin, followed by narirutin and myricetin, effectively interacted with the CoV Mpro protein. The Mpro demonstrated particularly efficient binding to the hesperidin flavonoid, followed by narirutin, isoquercetin, taxifolin, and petunidin.

Evaluation of silicon toxicity

The chosen flavonoid compounds were subjected to analysis using the SWISS-ADME database to enable the prediction of their toxicity. The findings of the absorbent analysis (A), which provided a summary of the ADME prediction, indicated that taxifolin, narirutin, hesperidin, baicalein, isoquercetin, myricetin, petunidin, and silibinin were predicted to exhibit high permeability (Table 1). The expression of low absorption in human intestinal absorption (HIA) value was less than 30%. The results indicated that the human gut's absorption of all flavonoid compounds was extensively investigated. Medications and other chemical substances that entered the body were eliminated by a transmembrane glycoprotein known as P-glycoprotein, which is involved in ATP binding [14,15]. The results of the study indicated that taxifolin, hesperidin, isoquercetin, myricetin, silibinin, and narirutin, which are nonsubstrate inhibitors of P-glycoprotein, exhibited either substrate or inhibitory properties toward P-glycoprotein. In the distribution (D) study, it was expected that the BBB permeability, with a logBBB value greater than 0.3, would have a high capacity to cross the blood-brain barrier [16]. All the flavonoids in this study were predicted to be BBB-permeable. CYP2D6 and CYP3A4 were the two crucial cytochrome P450 (CYP) liver enzyme subtypes in the metabolism (M) study [17]. The results demonstrated that taxifolin, narirutin, hesperidin, isoquercetin, myricetin, petunidin, and flavonoids did not support CYP2D6. Baicalein and silibinin were the only compounds that supported CYP2D6 and CYP3A4. These findings suggested that the liver might metabolize these flavonoids. The hepatocytes' basolateral membranes facilitate the absorption of drugs such as atorvastatin and metformin by using organic cation transporter 1 (OCT1), organic anion-carrying polypeptide 1B1 (OATP1B1), and organic cation transporter 1B3 (OATP1B3).

**Table 1:** Toxicity profile analysis of selected compounds.

Models	Compounds							
	Taxifolin	Narirutin	Hesperidin	Baicalein	Isoquercetin	Myricetin	Petunidin	Silibinin
Gastrointestinal absorption	High	Low	Low	High	Low	Low	High	Low
Blood-brain barrier permeation permeant	No	No	No	No	No	No	No	No
P-glycoprotein substrate	No	Yes	Yes	No	No	No	Yes	No
Cytochrome P1A2 inhibitor	No	No	No	Yes	No	Yes	Yes	No
Cytochrome P2C19 inhibitor	No	No	No	No	No	No	No	No
Cytochrome P2C9 inhibitor	No	No	No	No	No	No	No	No
Cytochrome P2D6 inhibitor	No	No	No	Yes	No	No	No	Yes
Cytochrome P3A4 inhibitor	No	No	No	Yes	No	Yes	No	Yes
Log Kp (skin permeation) cm/s	-7.48	-10.54	-10.12	-5.70	-8.88	-7.40	-7.71	-7.89

Molecular docking studies

**Figure 1:**
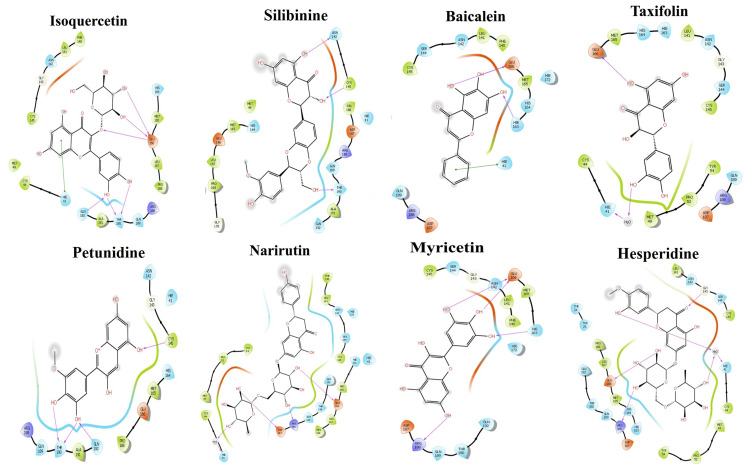
Molecular docking of main protease protein against selected ligands and two-dimensional (2D) interaction with the residues.

In the molecular docking experiments, the active residues in the binding cavity of proteins and ligand interactions were investigated for their known binding affinities. By adjusting the grid size to 3 Å, the active residues in the binding pocket of the protein (Mpro) were identified concerning the ligands. The docking score was considered to predict better interactions [18]. The flavonoids are listed in Table 2 based on the docking score. Based on the binding energy, the ranges for the eight individual flavonoids tested against Mpro were -3 to -15 kcal/mol, respectively. Isoquercetin had the highest docking score (-11.68 kcal/mol) against the Mpro complex out of all the examined compounds. Isoquercetin exhibited six bonds, including five hydrogen bonds (THR6854, CYS7007, and SER7074) and one pi-pi stacking (TRP6987) to the active residues (Figure 1; Table 2). Hesperidin (-15.73 kcal/mol) had the highest docking score for the Mpro, followed by narirutin and isoquercetin. Hesperidin and active residues of the major protease were shown to have about five active interactions. The Glu166 residue was a crucial amino acid implicated in the dimerization of Mpro and the development of the substrate binding pocket, and HIS41, GLU166, GLN189, and ARG188 residues were said to operate as active sites for the ligand binding site.

**Table 2:** Interactive profiles of flavonoid compounds with active site residues of target proteins.

Compound	Residue	Interaction	Type of bond
Main protease			
Hesperidin	Arginine (ARG) 7014	OH	H bond
	Glutamic acid (GLU) 7015	OH	H bond
	Threonine (THR) 6856	OH	H bond
Silibinin	Threonine (THR) 6856	OH	H bond
	Alanine (ALA) 6986	OH	H bond
Taxifolin	Cysteine (CYS) 7007	OH	H bond
Petunidin	Cysteine (CYS) 7007	OH	H bond
Narirutin	Threonine (THR) 6854	OH	H bond
	Asparagine (ASN) 6853	OH	H bond
	Arginine (ARG) 7053	OH	H bond
Isoquercetin	Threonine (THR) 6854	OH	H bond
	Threonine (THR) 6854	OH	H bond
	Tryptophan (TRP) 6987	-	Pi-Pi
	Cysteine (CYS) 7007	OH	H bond
	Serine (SER) 7074	OH	H bond
Baicalein	Cysteine (CYS) 7007	OH	H bond
Myricetin	Cysteine (CYS) 7007 Tryptophan (TRP) 6987	OH	H bond Pi-Pi
	Threonine (THR) 6856	OH	H bond
	Serine (SER) 7074	OH	H bond

MD simulations 

**Figure 2:**
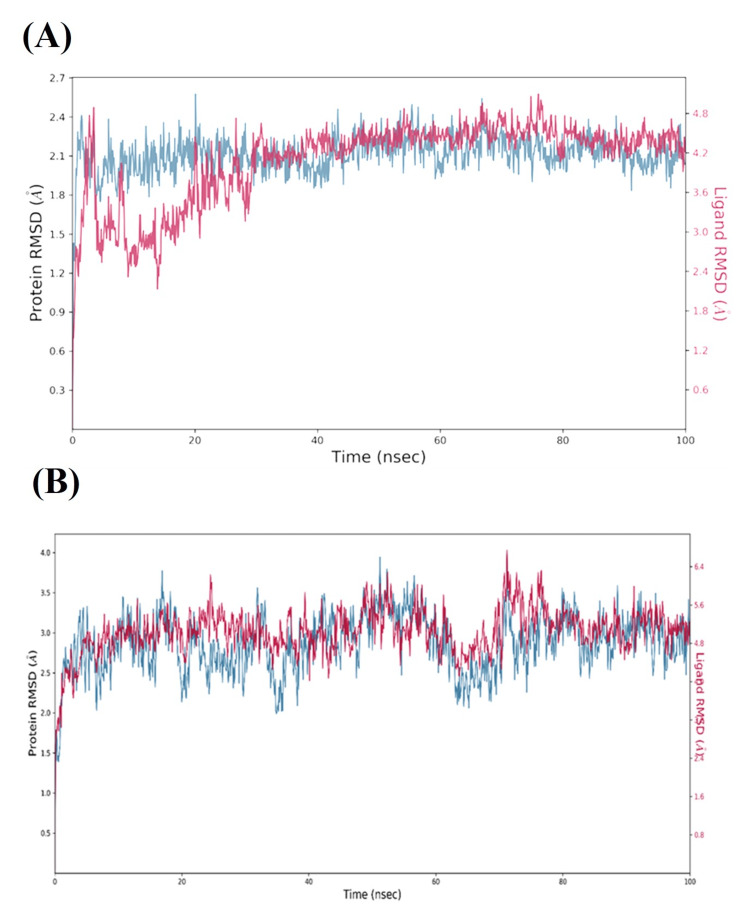
Analysis of molecular dynamics for selected compounds (A) isoquercetin and (B) narirutin (main protease) RMSD at 100 ns. RMSD, root mean square deviation

The highest docked complexes (isoquercetin and narirutin) received 100 ns of MD simulations. The findings of the MD simulations demonstrated that the ligand binds steadily to the active site residues Mpro. Figure 2 displays the root-mean-square deviations (RMSDs) of the backbone atoms of the Mpro and associated ligands. The docking experiments based on interactions with residues such as His41, His 164, Glu166, Asp187, Thr190, and Gln192 demonstrated minor RMSD variations in the MD results of Mpro residues. It suggests that both drugs (isoquercetin and narirutin) were closer to the Mpro active site and had a stronger affinity. Similar to this, the MD findings revealed low RMSD variations in Mpro residues in connection with docking residues such as Asn6853, Thr6854, Thr6856, Trp6987, and Cys7007 [19].

Analysis of DFT

**Figure 3:**
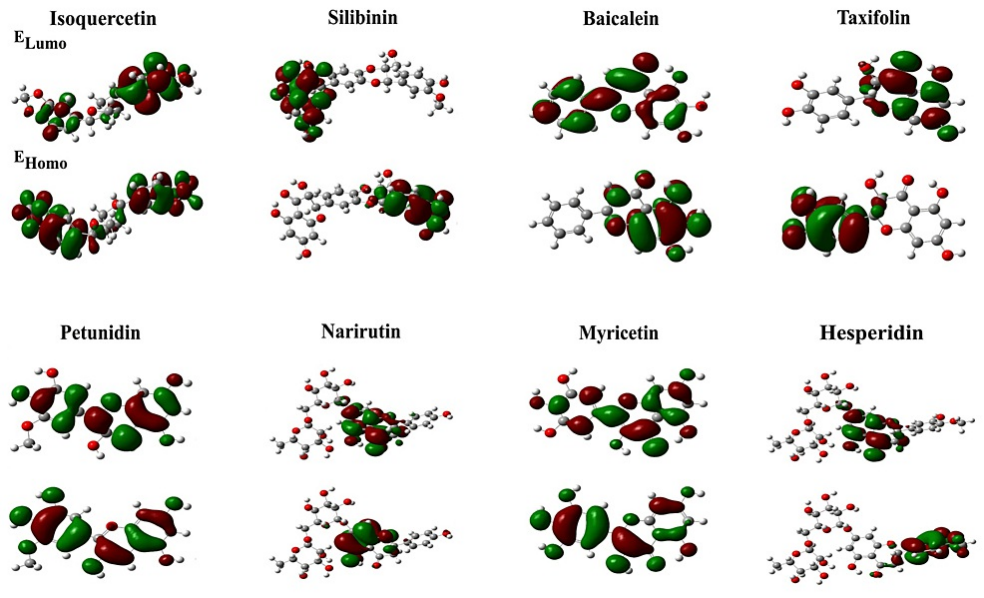
Analysis of density functional theory of selected compounds.

The DFT calculations in the gas phase were performed using the B3LYP-311 G (d,p) method. A molecule with a higher HOMO value has a more potent electron donor, whereas one with a lower HOMO value has a weaker electron acceptor. Additionally, intermolecular charge transfer and molecular bioactivity have been significantly impacted by the smaller energy gap between LUMO and HOMO energies. Therefore, a significant energy difference in the hit molecules harms the electron's transition from HOMO to LUMO. It eventually contributes to the Mpro inhibitor's low affinity. The following electronic band gap values were obtained: 0.20525 a.u. for isoquercitrin, 0.13454 a.u. for silibinin, 0.13994 a.u. for baicalein, 0.12717 a.u. for taxifolin, 0.05562 a.u. for petunidin, 0.17683 a.u. for narirutin, 0.1287 a.u. for myricetin, and 0.13768 a.u. for hesperidin (Figure 3). The cloud density of the HOMO and LUMO boundary orbital states was established in the red and green regions, respectively. Recent studies have demonstrated that frontier molecular orbitals must be considered when examining organized activity relations [20].

Analysis of binding free energy (MMPBSA)

The interaction-binding free energy (MMPBSA) between the Mpro protein and flavonoid compounds was calculated using Desmond software. Additionally, per-residue energy decomposition (MMGBSA) was analyzed from the MD simulations. The binding free energy for individual components, namely, H-bond, coulomb, and Van der Waals forces, was calculated using the Desmond software tool (MMPBSA/MMGBSA). Table 3 shows that these values were attributed to energy bonding with flavonoid compounds and CoV proteins. The Gibbs binding free energy for the interaction between flavonoid compounds and Mpro proteins was recorded as -32.13 kcal/mol for narirutin and -36.37 kcal/mol for isoquercetin [21-23].

**Table 3:** Analysis of binding free energy for the main protease (Mpro) protein.

Contribution (kcal/mol)	Isoquercetin	Hesperidin	Narirutin
Binding free energy (Δ*G*_bind_)	-31.97 ± 0.18	-36.37 ± 0.27	-32.13 ± 0.20
Coulomb energy (Δ*G*_coulomb_)	-20.52 ± 0.11	-42.36 ± 0.29	-31.13 ± 0.41
Covalent binding energy (Δ*G*_covalent_)	8.35 ± 0.32	8.65 ± 0.27	7.64 ± 0.18
Hydrogen binding energy (Δ*G*_H-bond_)	-8.71 ± 0.48	-10.53 ± 0.31	-9.51 ± 0.20
Lipophilicity free energy (Δ*G*_lipo_)	-4.25 ± 0.26	-6.95 ± 0.15	-4.05 ± 0.10
Packing free energy (Δ*G*_packing_)	-4.62 ± 0.19	-5.32 ± 0.23	-4.13 ± 0.14
Total polar energy (Δ*G*_GB_)	35.27 ± 0.23	39.71 ± 0.28	40.12 ± 0.33
Hydrophobic binding energy (Δ*G*_vdW_)	-11.40 ± 0.34	-13.83 ± 0.21	-14.53 ± 0.14

## Discussion

The medicinal plant-derived bioactive compounds have demonstrated a variety of pharmacological and biological activities, including antiproliferative, anti-inflammatory, antiatherogenic, antiallergic, antimicrobial, immunomodulatory, and antiviral properties for human health [9,10]. They are also nontoxic, natural compounds that are friendly to the environment. Flavonoid chemicals from medicinal plants have been shown to have antiviral action against the H1N1, Ebola, influenza, and dengue viruses [10]. One of the crucial novel therapeutic strategies for viral infections and an inhibitor of viral proteases is flavonoid chemicals found in natural medicinal plants [11]. The benefits of flavonoids include being active even at low medication concentrations, being highly accessible to nature, being varied, and having no or very few negative effects. The main resource for discovering and developing a large number of new potential antiviral medicines are natural medicinal herbs that modulate flavonoids [12,13]. The group of phenyl benzopyrones, known as flavonoids, has a low molecular weight and many pharmacological effects, such as anti-inflammatory, antiviral, and antioxidant activities [14,15]. Flavonoids inhibit the release of viral particles from being executed, which disrupts viral replication. Their effectiveness in reducing viral amplification in the human body [16,17]. The process of medication excretion is subject to the effect of two key factors, namely, the hydrophilicity and molecular weight of the medicine [24,25]. 

As reported by Jain et al. [26], 10 natural flavonoid compounds were analyzed for docking scores with the spike protein of SARS-CoV-2 virus, including apigenin at -7.8 kcal/mol, chrysin at −8.1 kcal/mol, fisetin at -8.3 kcal/mol, galangin at -8.2 kcal/mol, hesperetin at -7.7 kcal/mol, luteolin at -8.0 kcal/mol, morin at -8.1 kcal/mol, naringin at -9.8 kcal/mol, quercetin at -8.2 kcal/mol, rutin at- 9.2 kcal/mol, and dexamethasone at -7.9 kcal/mol. Molecular simulation analysis was conducted on 10 flavonoid compounds, and their stability was determined at 0.2 to 0.4 ns.

Imran et al. [27] investigated the flavonoid compounds' activity against SARS-CoV-2 Mpro protein and docking score, namely, -9.9 kcal/mol of amentoflavone, -8.0 kcal/mol of kaempferol, -8.0 kcal/mol of baicalein, -7.6 kcal/mol of catechin, -8.2 kcal/mol of naringin, -7.7 kcal/mol of naringenin, -7.8 kcal/mol of galangin, -8.1 kcal/mol of kuwanon C, -8.0 kcal/mol of morin, -8.3 kcal/mol of morusin, -8.3 kcal/mol of scutellarein, -8.2 kcal/mol of wogonin, -8.3 kcal/mol of apigenin, and -8.0 kcal/mol of fisetin, respectively.

The primary transporters responsible for the elimination of cationic medications into the urine are organic cation transporters (OCTs) and multi-drug- and toxin-extrude proteins (MATEs) [4]. The findings of this study indicate that the flavonoids examined in this study are not expected to hinder the activity of MATE-1, OCT-1, and OCT-2. This suggests that the flavonoids have a safe disposal profile. The findings from the experiment on toxicity (T) indicated that all quinolones have induced ocular corrosion and carcinogenicity. In their study, Imran et al. [26] showed that amentoflavone, kaempferol, baicalein, catechin, naringin, naringenin, galangin, kuwanon C, morin, morusin, scutellarein, wogonin, apigenin, and fisetin flavonoids had good intestinal absorption, with absorption rates between 96% and 98%. The low absorption rate of the molecules is correlated with their high molecular weight. Ninety-seven percent of the high-molecular-weight amentoflavone compound was absorbed in the intestine. According to our test, flavonoids are solubilized in water between -1 and -5, with the aqueous solubility of flavonoids ranging from 3.78 to 2.77, which is moderate to high. Finally, the results of the prediction analysis revealed that the ADMET properties of flavonoids closely resemble those of established anti-CoV and anti-SARS-CoV-2 drugs. It provides evidence to consider the repurposing of specific pharmaceuticals for the therapeutic management of CoV. Flavonoids have been observed to inhibit the function of drug transporters, leading to a decrease in the efficacy of pharmaceutical substances, thereby giving rise to drug-drug interactions that hold therapeutic significance [26,27]. Thus, this study investigated the effects of taxifolin, narirutin, hesperidin, baicalein, isoquercetin, myricetin, petunidin, and silibinin. The outcome demonstrated that all the chosen flavonoids seemed to be OATP1B1-OATP1B3 inhibitors.

According to Imran et al. [26], Mpro complexes with Amentoflavone and morusin were simulated for 100 ns with the stability and fluctuation of the receptor-ligand complexes analyzed during simulations, and a backbone RMSD was used to calculate the resulting trajectory. Throughout the entire simulation, the protein-ligand complex remained stable. During the first 10 ns, a few fluctuations in protein were seen, but then it remained stable for 90 ns. Between Thr24 and Leu50, Asn142 and Cys145, His163 and Thr169, and Val186 to Gln192, 27 ligand contacts were observed with residues in proteins. In addition to hydrogen bonds, these residues also formed ionic bonds, hydrogen bonds, hydrophobic bonds, and water bridges. The active residues of ligands are in hydrophobic interaction with the polar-forming ligands, resulting in ligand stability.

Limitations and future recommendations

This study marks a promising start for developing plant-based antiviral compounds; however, inherent limitations must be acknowledged. A predictive model and a computational analysis were used to obtain the results of our study. Consequently, experimental validation is crucial since it provides concrete evidence of a compound's safety and effectiveness.

## Conclusions

The COVID-19 Mpro complex proteins were docked with eight compounds. Mpro showed high docking scores on hesperidin, narirutin, and isoquercetin. This study evaluated essential antiviral action from plant-mediated flavonoid compounds. These flavonoids could be used as antiviral medications to control the CoV. As a result, plant flavonoid derivatives may shortly produce a fresh development in synthesizing natural drugs and various biological applications
